# Norgestimate inhibits staphylococcal biofilm formation and resensitizes methicillin-resistant *Staphylococcus aureus* to β-lactam antibiotics

**DOI:** 10.1038/s41522-017-0026-1

**Published:** 2017-07-21

**Authors:** Yutaka Yoshii, Ken-ichi Okuda, Satomi Yamada, Mari Nagakura, Shinya Sugimoto, Tetsuo Nagano, Takayoshi Okabe, Hirotatsu Kojima, Takeo Iwamoto, Kazuyoshi Kuwano, Yoshimitsu Mizunoe

**Affiliations:** 1 Department of Bacteriology, The Jikei University School of Medicine, 3-25-8 Nishi-Shimbashi, Minato-ku, Tokyo, 105-8461 Japan; 2 Jikei Center for Biofilm Science and Technology, 3-25-8 Nishi-Shimbashi, Minato-ku, Tokyo, 105-8461 Japan; 3 Division of Respiratory Diseases, Department of Internal Medicine, The Jikei University School of Medicine, 3-25-8 Nishi-Shimbashi, Minato-ku, Tokyo, 105-8461 Japan; 4 Drug Discovery Initiative, The University of Tokyo, 7-3-1 Hongo, Bunkyo-ku, Tokyo, 113-0033 Japan; 5 Division of Molecular Cell Biology, Core Research Facilities for Basic Science, The Jikei University School of Medicine, 3-25-8 Nishi-Shimbashi, Minato-ku, Tokyo, 105-8461 Japan

## Abstract

Formation of bacterial biofilms on medical devices can cause severe or fatal infectious diseases. In particular, biofilm-associated infections caused by methicillin-resistant *Staphylococcus aureus* are difficult to eradicate because the biofilm is strongly resistant to antibiotics and the host immune response. There is no effective treatment for biofilm-associated infectionss, except for surgical removal of contaminated medical devices followed by antibiotic therapy. Here we show that norgestimate, an acetylated progestin, effectively inhibits biofilm formation by staphylococcal strains, including methicillin-resistant *S. aureus*, without inhibiting their growth, decreasing the selective pressure for emergence of resistance. 17-Deacetyl norgestimate, a metabolite of norgestimate, shows much weaker inhibitory activity against staphylococcal biofilm formation, indicating that the acetyl group of norgestimate is important for its activity. Norgestimate inhibits staphylococcal biofilm formation by inhibiting production of polysaccharide intercellular adhesin and proteins in the extracellular matrix. Proteome analysis of *S*. *aureus* indicated that norgestimate represses the expression of the cell wall-anchored protein SasG, which promotes intercellular adhesion, and of the glycolytic enzyme enolase, which plays a secondary role in biofilm formation. Notably, norgestimate induces remarkable changes in cell wall morphology, characterized by increased thickness and abnormal rippled septa. Furthermore, norgestimate increases the expression level of penicillin binding protein 2 and resensitizes methicillin-resistant *S. aureus* to β-lactam antibiotics. These results suggest that norgestimate is a promising lead compound for the development of drugs to treat biofilm-associated infections, as well as for its ability to resensitize methicillin-resistant *S. aureus* to β-lactam antibiotics.

## Biofilm formation: Benefits of selective inhibition

A synthetic molecule related to the hormone progesterone might keep medical devices free of biofilms without promoting antibiotic resistance. Implanted devices that have become contaminated with biofilms generally must be surgically removed prior to treating the underlying infection with antibiotics. Ken-ichi Okuda and colleagues at The Jikei University School of Medicine in Tokyo, with co-workers elsewhere in Japan, found that the synthetic progesterone analog norgestimate inhibits biofilm formation without inhibiting bacterial growth. They regard this selective effect on biofilm formation as a significant advantage, as it reduces the risk of inducing resistance in the targeted bacteria. They demonstrated the effect using staphylococcal bacteria, including the problematic and highly dangerous methicillin-resistant *Staphylococcus aureus* (MRSA). The research also indicated that norgestimate can resensitize MRSA bacteria to some of the antibiotics they are resistant to.

## Introduction

*Staphylococcus aureus* is a gram-positive bacterium that can cause life-threatening community-acquired and nosocomial-associated infections. Methicillin-resistant *S*. *aureus* (MRSA), which is resistant to β-lactam antibiotics such as penicillins, cephalosporins, and carbapenems, is among the most widely distributed drug-resistant bacterial species worldwide. Resistance to methicillin is primarily conferred by the acquisition of *mecA*, which encodes penicillin-binding protein 2a (PBP2a), which has a low affinity for β-lactam antibiotics.^1^ In the United States, 19,000 hospitalized patients die annually because of MRSA infections.^2^

Biofilm formation is an important *S. aureus* virulence factor, characterized by attachment of multilayered cells to abiotic and biotic surfaces. Cells in the biofilm are embedded in an extracellular matrix (ECM) comprising extracellular DNA, proteins, and polysaccharide intercellular adhesin (PIA).^3^ Biofilm cells are more resistant to antibiotics than planktonic cells because their metabolic activity is lower, and cell division, which is required for sensitivity to many antibiotics, is markedly attenuated.^4, 5^ Thus, biofilm cells can withstand exposure to antibiotics at concentrations up to 1000-fold higher than those required to inhibit the growth of planktonic cells.^6^

Staphylococci, including MRSA, are major pathogens of biofilm-associated infections (BAIs) caused by contamination of medical devices, such as catheters, pacemakers, and prosthetic joints.^7^ Because of their high resistance to antibiotics, BAIs are difficult to treat.^8^ Indeed, among patients with hospital-acquired *S. aureus* bacteremia associated with medical devices, the 12-week mortality range is 17–35%.^9^ Thus, BAIs are usually treated by surgical removal of the contaminated devices, followed by antibiotic therapy.^10^ However, surgical removal of devices can be risky, and can lead to complications or even fatality. Therefore, it is imperative to develop alternative treatments for BAIs.

Although the development of new antibiotics represents an important strategy to combat drug-resistant bacteria, there is a risk that new drug-resistant bacteria with novel antibiotic-resistance mechanisms will emerge and spread. Here we aimed to identify compounds that inhibit staphylococcal biofilm formation without significantly affecting bacterial growth and viability because such compounds will likely exert low pressure to select for antibiotic resistance.

## Results

### Norgestimate (NGM) inhibits staphylococcal biofilm formation

**Fig. 1:**
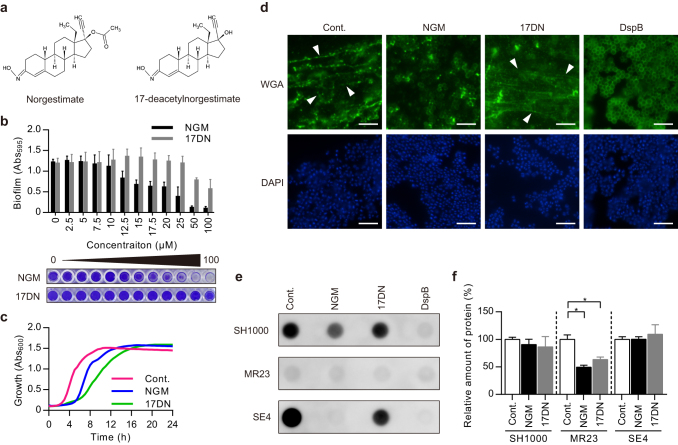
Staphylococcal biofilm inhibitory activities of norgestimate (NGM) and 17-deacetylnorgestimate (17DN). **a** Structures of NGM and 17DN. **b** MR23 biofilm-inhibitory activities of NGM and 17DN. Data are presented as the mean ± SD (standard deviation) (*n* = 3). The graphs represent the average absorbance of the crystal violet-stained biofilms formed on 96-well plates. Photographs of representative wells are shown below the graphs. **c** Effects of NGM and 17DN on the growth of MR23. Cells were cultured in BHI broth under shaking conditions, with or without 50 μM of the test compound. **d** Fluorescence microscopy of PIA in a SH1000 biofilm. PIA and DNA were stained using WGA-Alexa488 (WGA; *upper panels*) and DAPI (*lower panels*), respectively. *Arrowheads* indicate the filamentous structural PIA. Scale bars = 5 μm. **e** Dot blot assay of PIA. **f** Quantification of ECM proteins. Data are presented as the mean ± SD (*n* = 3). *p < 0.01. In **d**–**f**, cells were cultured under biofilm-forming conditions, with or without 50 μM of the test compound, or 20 μg/mL of DspB. SH1000, *S. aureus* SH1000; MR23, *S. aureus* MR23; SE4, *S*. *epidermidis* SE4. *Cont*. control, *NGM* norgestimate, *17DN* 17-deacetylnorgestimate, *DspB* dispersin B

High-throughput screening of 50,000 compounds identified NGM, which is a synthetic progestin of the 19-norsteroid series, as an inhibitor of staphylococcal biofilm formation (Fig. 1a). NGM is used for oral contraception, treatment of hyperandrogenism in women, and hormone replacement therapy.^11^ NGM inhibited biofilm formation in all staphylococcal strains tested (eight strains of *S. aureus* and two strains of *Staphylococcus epidermidis*), with IC_50_ values ranging between 12.0 and 22.5 μM (Table 1, Table S1, Fig. 1b). In contrast, the IC_50_ value of 17-deacetyl NGM (17DN) (Fig. 1a), which is a deacetylated metabolite of NGM generated in the liver and intestines,^11^ was at least 3.1-fold higher in each strain (3.1–6.1-fold in *S*. *aureus* and >6.6-fold in *S*. *epidermidis*) (Table 1, Fig. 1b), suggesting that the acetyl group of NGM is important for biofilm inhibition. Then we analyzed the effects of NGM and 17DN on the growth of *S*. *aureus*. We found that NGM and 17DN prolonged the lag phase compared with the control, by unknown mechanisms. However, neither compound significantly affected the final cell density after 24 h of incubation (Fig. 1c).

**Table 1:** Biofilm inhibitory activities of NGM and 17DN against various staphylococcal strains

Strain	IC_50_^*^ value (μM)	
	NGM^a^	17DN^b^
MSSA		
MS3	22.5	92.2
MS4-5	22.4	78.5
MS18	13.1	75.6
SH1000	15.6	49.0
MRSA		
MR2	13.4	76.5
MR4	13.8	84.0
MR11	12.0	62.7
MR23	15.9	95.6
*Staphylococcus epidermidis*		
SE4	15.1	>100
SE21	14.6	>100

^*^Half maximal inhibitory concentration

^a^ Norgestimate

^b^ 17-deacetyl norgestimate

Next, we compared the biofilm inhibitory activities of NGM against staphylococcal strains with those of previously reported biofilm inhibitors. We tested aryl rhodanines (MBX-1240, MBX-1246, and MBX-1384),^12^ benzimidazole (ABC-1),^13^ D-tyrosine,^14^ and tannic acid.^15^ Among the tested biofilm inhibitors, ABC-1, D-tyrosine, and tannic acid did not inhibit biofilm formation under our experimental conditions (IC_50_ > 100 µM) (Table S2). Against *S*. *aureus*, NGM showed greater inhibition of biofilm formation than MBX-1240, and almost the same inhibitory activity as MBX-1246 and MBX-1384. Notably, NGM inhibited biofilm formation of *S*. *epidermidis* more strongly than the three aryl rhodanines (>4.3-fold in SE4 and >3.3-fold in SE21) (Table S2).

### NGM inhibits ECM production by staphylococci

The ECM plays an important role in the formation of biofilms by staphylococci.^3^ Therefore, we reasoned that NGM might affect the production of ECM components. We cultured the methicillin-susceptible *S. aureus* (MSSA) laboratory strain SH1000, the clinical MRSA isolate MR23, and the clinical isolate *S*. *epidermidis* SE4 with or without NGM under conditions suitable for biofilm formation. We measured extracellular PIA accumulation by staining with wheat germ agglutinin (WGA)-Alexa 488. NGM clearly decreased the number of filamentous structures in cultures of SH1000 and SE4 (Fig. 1d, Fig. S1). These structures were identified as PIA because they disappeared after treatment with dispersin B (DspB), which is a PIA-degrading β-*N*-acetylglucosaminidase.^16^ Further, a dot blot assay showed that NGM significantly decreased the amount of PIA in the ECM of SH1000 and SE4 (Fig. 1e, Fig. S2a, c).

The ability of NGM to inhibit biofilm formation by SH1000 and SE4 thus appears to be dependent on inhibition of PIA production. In contrast, MR23 did not produce PIA, regardless of NGM presence (Fig. 1e, Fig. S1, Fig. S2b). MR23 is known to form a proteinaceous biofilm.^17, 18^ Therefore, we speculated that NGM inhibits biofilm formation in MR23 via a mechanism that differs from that responsible for the inhibition of PIA production. Using a fluorescent probe, we quantified proteins in ECM preparations extracted from each strain and found that treatment with NGM decreased the protein quantity in ECM extracts of MR23 cultures (Fig. 1f).

### NGM inhibits the expression of proteins involved in biofilm formation

**Fig. 2:**
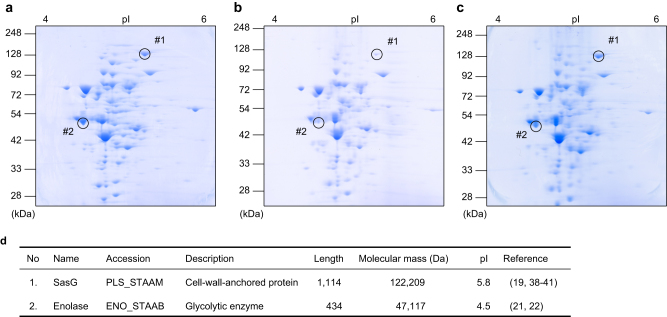
The effects of NGM on the proteome of *S. aureus*. **a**–**c** Two-dimensional (2-D) electrophoresis in the presence or absence of NGM or 17DN (**a** DMSO control; **b** NGM; **c** 17DN). **d** Identification of proteins, whose expressions were decreased in the presence of NGM on 2-D gel

Next, we investigated the effect of NGM on the protein expression profile of *S. aureus* MR23 by two-dimensional (2-D) gel electrophoresis (Fig. 2a–c). Following comparison of protein expression in the 2-D gel images, two protein spots (#1 and #2 in Fig. 2) that showed decreased expression in the presence of NGM but not 17DN were subjected to further protein identification analysis. LC-MS/MS analysis identified proteins #1 and #2 as surface protein G (SasG) and enolase, respectively (Fig. 2d).

The cell wall–anchored protein SasG promotes intercellular adhesion during the accumulation phase of biofilm formation in *S. aureus*, independent of PIA.^19, 20^ We also verified the expression of *sasG* at the transcriptional level using microarray and real-time polymerase chain reaction (PCR) assays (Table S3). In contrast to the results of protein expression, the expression of *sasG* was enhanced at the transcriptional level in the presence of NGM or 17DN. This suggests that NGM affects the expression of SasG at the translational or post-translational level.

**Fig. 3:**
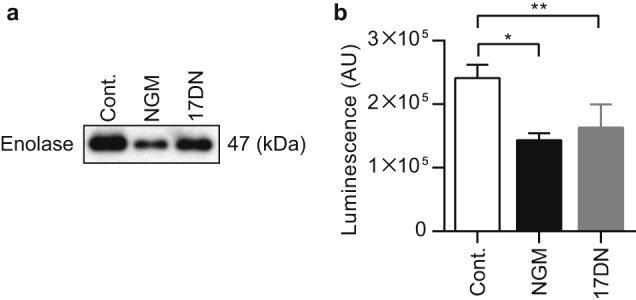
The effects of NGM on enolase expression in the ECM. **a** Western blotting analysis of enolase expression in the ECM using an anti-enolase antibody. **b** Band intensities of western blotting analysis of enolase expression in the ECM. Data are presented as the mean ± SD (*n* = 3). *p < 0.01, **p < 0.05

Enolase, which converts 2-phosphoglyceric acid to phosphoenolpyruvate in the glycolytic pathway, associates with the bacterial cell surface and mediates host factor attachment.^21, 22^ We investigated whether NGM affects the level of enolase in the ECM using western blotting. NGM reduced the protein level of enolase in the ECM compared to the control (Fig. 3). Moreover, microarray and real-time PCR assays revealed that transcription of the gene encoding enolase was downregulated in the presence of NGM (Table S3).

### NGM disrupts cell wall homeostasis

**Fig. 4:**
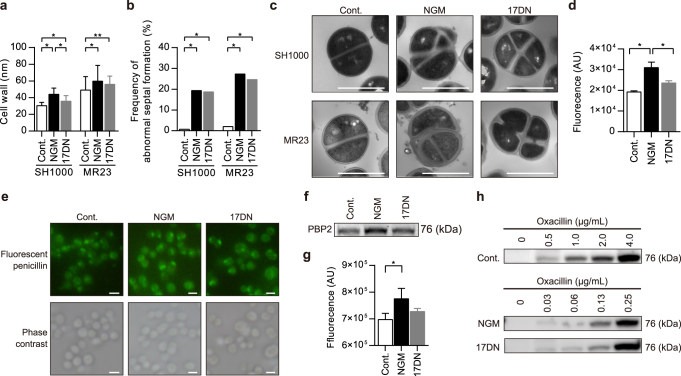
The effects of NGM on cell morphology and expression of penicillin binding protein (PBP) 2 and PBP2a. **a** Cell wall thickness of MR23 and SH1000 cells treated with NGM or 17DN for 24 h. Data are presented as the mean ± SD (*n* = 50). *p < 0.01, **p < 0.05. **b** The frequency of abnormal septal formation in cells treated with NGM or 17DN for 24 h. *p < 0.01. Data are presented as the mean (*n* = 150). **c** Transmission electron microscopy (TEM) images. Representative cells with abnormal septal formation are shown (NGM and 17DN). Scale bars = 1 μm. **d** Fluorescence intensities of cells treated with FL-penicillin. Data are presented as the mean ± SD (*n* = 3) *p < 0.05. **e** Fluorescence microscopy of cell-associated FL-penicillin. Scale bars = 1 μm. **f** SDS-PAGE analysis of PBP2 detected using FL-penicillin. **g** Fluorescence intensities of the PBP2 bands detected using FL-penicillin. Data are presented as the mean ± SD (*n* = 3) *p < 0.05. **h** Western blotting analysis of PBP2a expression using an anti-PBP2a antibody. In these experiments, cells were cultured under biofilm-forming conditions, with or without 50 μM of the test compound

Transmission electron microscopy (TEM) revealed that NGM induced a significant increase in the cell wall thickness of MR23 and SH1000 cells (Fig. 4a). 17DN induced a moderate increase in cell wall thickness. The thickness of the MR23 cell walls was significantly increased in the presence of NGM at 6, 12, and 24 h of culture (Fig. S3a). The frequency of abnormal septal formation was also increased in MR23 and SH1000 cells treated with NGM or 17DN compared to the control cells (Fig. 4b, c, Fig. S3b). We then analyzed the expression levels of genes related to peptidoglycan turnover. Microarray and real-time PCR assays revealed that the expression levels of certain genes related to peptidoglycan synthesis (*femA*, *femC*, and *pbp2*) and hydrolysis (*atl, isaA*, and *lytM*) increased more than 2-fold in the presence of NGM (Table S4). Altered expression of these genes seems to be involved in the formation of abnormal cell walls, because the balance between peptidoglycan synthesis and hydrolysis stringently regulates bacterial cell wall homeostasis.^23^

### NGM resensitizes MRSA to β-lactam antibiotics

We next used fluorescently-labeled (FL)-penicillin to determine the expression level and localization of PBPs in the presence and absence of NGM. Cells cultured with NGM emitted significantly stronger fluorescence than control cells or cells cultured with 17DN (Fig. 4d, e), indicating that increased amounts of FL-penicillin localized to the surface of cells cultured with NGM. Next, after forming complexes of FL-penicillin and PBPs in *S. aureus* MR23, we used SDS-PAGE to estimate the expression level of PBPs. NGM treatment increased the expression level of PBP2 (Fig. 4f, g, Fig. S4a), consistent with the results of the microarray and real-time PCR assays (Table S4). PBPs are involved in the final step of peptidoglycan synthesis and are targets of β-lactam antibiotics.^24^ Therefore, we investigated the influence of NGM on the antibiotic susceptibility of the clinical isolates MR2, MR4, MR11, and MR23 under the standard conditions for the determination of the minimum inhibitory concentration (MIC) recommended by the Clinical & Laboratory Standards Institute (Mueller–Hinton) and for the biofilm conditions [brain heart infusion (BHI) broth supplemented with 1% glucose: BHIG]. The data indicate that NGM resensitized all MRSA strains to the β-lactam antibiotics oxacillin, ampicillin, cefazolin, cefmetazole, flomoxef, cefoxitin, and imipenem, under both culture conditions (Table 2, Table S5, Table S6). A 2- to 64-fold increase in susceptibility to the β-lactam antibiotics was observed, although the degree of resensitization varied between strains, antibiotics, and culture conditions. 17DN moderately resensitized most strains.

**Table 2:** The effects of NGM on antibiotic susceptibility of *S. aureus* MR23 in Mueller–Hinton or BHIG broth

Antibiotic	Mueller–Hinton			BHIG		
	MIC^*^ (μg/mL)			MIC (μg/mL)		
	Control	NGM^a^	17DN^b^	Control	NGM	17DN
β-lactam						
Oxacillin	32	4	4	8	0.5	0.5
Ampicillin	8	0.25	4	4	0.25	0.25
Cefazolin	16	1	4	4	1	1
Cefmetazole	16	8	16	8	2	2
Flomoxef	8	1	4	4	1	1
Cefoxitin	16	8	16	16	8	8
Imipenem	2	0.25	0.25	0.25	0.03	0.06
Glycopeptide						
Vancomycin	2	1	1	2	2	2
Teicoplanin	2	1	1	2	1	1
Oxazolidinone						
Linezolid	2	2	2	4	2	2
Aminoglycoside						
Gentamicin	4	2	2	>8	>8	>8
Arbekacin	0.5	0.25	0.25	2	1	1
Tetracycline						
Minocycline	<2	<2	<2	<2	<2	<2
Macrolide						
Erythromycin	0.25	0.25	0.25	0.5	0.5	0.5
Lincomycin						
Clindamycin	<0.06	<0.06	<0.06	0.12	0.12	0.12
Fosfomycin						
Fosfomycin	<32	<32	<32	32	64	64
Fluoroquinolone						
Levofloxacin	>4	>4	>4	4	4	4
Sulfonamide						
Sulfamethoxazole–Trimethoprim	<10	<10	<10	40	20	20

In these experiments, cells were cultured under biofilm-forming conditions, with 50 μM of the test compound

^*^Minimum inhibitory concentration

^a^ Norgestimate

^b^ 17-deacetyl norgestimate

PBP2a is required for resistance of MRSA to β-lactam antibiotics.^25^ We therefore determined the effects of NGM on the expression of PBP2a. *S. aureus* MR23 was cultured in the presence of NGM, 17DN, or oxacillin at concentrations below the MIC, and expression levels of PBP2a were analyzed by western blotting. In the absence of oxacillin, expression of PBP2a was not detected under any conditions. Unexpectedly, in the presence of NGM and 17DN, PBP2a expression was induced at lower concentrations of oxacillin than the control (Fig. 4h, Fig. S4b–d). These results indicate that NGM and 17DN enhanced β-lactam-inducible expression of PBP2a despite their β-lactam resensitizing effects. Notably, as with MRSA, NGM and 17DN increased the susceptibility of MSSA strains, which lack PBP2a, to the β-lactam antibiotics (Table S7, Table S8). Therefore, NGM and 17DN were shown to induce the resensitization to β-lactam antibiotics regardless of the presence of PBP2a.

## Discussion

This study demonstrated that NGM inhibits the formation of biofilms by various staphylococcal strains. NGM is an acetylated progestin that is used as an oral contraceptive. To our knowledge, there is no report demonstrating that progestins inhibit bacterial biofilm formation. NGM is swiftly converted in the liver and intestines to 17DN, a deacetylated metabolite with progestogenic properties.^11^ Because our study indicated that the acetyl group of NGM is important for biofilm inhibition (Fig. 1a, b), the clinical application of NGM as a therapeutic agent for BAIs will require chemical modifications that improve its pharmacokinetics and potency. Alternatively, NGM itself may serve as a topical drug, used from the initial stage of wound infection, to prevent the deterioration of biofilm-associated wound infections. Moreover, NGM and 17DN are considered less likely to induce the emergence of resistant bacteria, because compounds that do not significantly affect bacterial growth exert little or no selective pressure.

ECM components provide mechanical stability to biofilms and mediate their adhesion to surfaces.^26^ PIA, which is synthesized from *N*-acetylglucosamine (GlcNAc) by enzymes encoded by the intercellular adhesion operon, is a major ECM component that mediates intercellular adhesion in biofilms formed by certain staphylococcal strains.^27–29^ Further, PIA contributes to the structural integrity of biofilms during the accumulation phase of biofilm formation.^30^ NGM significantly inhibited PIA production by SH1000 and SE4 (Fig. 1d, e, Fig. S1, Fig. S2). We inferred that the decreased levels of PIA observed in the presence of NGM were associated with abnormal cell wall synthesis. Peptidoglycan synthesis requires uridine diphosphate *N*-acetylglucosamine (UDP-GlcNAc) as a subunit component.^31, 32^ Increased cell wall synthesis in the presence of NGM might lead to depletion of UDP-GlcNAc, which is also a substrate for PIA.^27, 33, 34^ Although the PIA inhibitory effect is considered to be involved in NGM-mediated inhibition of biofilm formation by PIA-producing staphylococcal strains, it was indicated that PIA is dispensable for biofilm formation in many strains.^35^ It has been reported that proteins play an important role in PIA-independent biofilm formation.^22, 36, 37^ Our data indicate that NGM inhibits protein-dependent biofilm formation by repressing the expression of proteins that localize at the cell surface and contribute to biofilm formation (Figs. 1f, 2, 3). SasG, a well-studied, cell wall-anchored protein of *S*. *aureus*, is covalently attached to peptidoglycan via a C-terminal cell wall-anchoring domain.^19, 20^ SasG contains a stretch of tandemly arrayed B repeats, which promote cell-to-cell accumulation during biofilm formation in a Zn^2+^-dependent manner.^38–41^ In contrast, enolase is a cytoplasmic protein that functions as a glycolytic enzyme. Recent reports have suggested that some cytoplasmic proteins, including enolase, are released from the cell and become associated with the cell surface, serving as “moonlighting components” of the ECM.^42, 43^ Suppression of the expression of two different types of proteins, a cell wall-anchored protein, SasG, and a moonlighting protein, enolase, may contribute to the potent and broad biofilm inhibitory activity of NGM.

NGM resensitized all MRSA strains and increased the susceptibility of all MSSA strains to β-lactam antibiotics (Table 2, Tables S5–S8). PBP2a is required for resistance of MRSA to β-lactam antibiotics.^25^ We therefore anticipated that NGM might repress the expression of PBP2a. Expression of *mecA*, which encodes PBP2a, has been reported to rely on a three-component system consisting of a transcriptional repressor (MecI), a β-lactam sensing inducer (MecR1), and an antirepressor (MecR2).^44^ Contrary to our expectations, western blotting suggested that NGM and 17DN enhanced β-lactam-inducible PBP2a expression, despite their β-lactam resensitizing effects (Fig. 4h, Fig. S4b–d). This implies that in the presence of NGM, PBP2a-mediated β-lactam resistance is not effective. Although the detailed mechanism of resensitization to β-lactam antibiotics is not yet clear, abnormal cell wall synthesis involving increased expression of PBP2 appears to affect the sensitivity to these antibiotics (Fig. 4a–c, f, g, Fig. S3).

In conclusion, NGM inhibited the production of PIA and ECM component proteins that are important for biofilm formation by each staphylococcal strain, thereby inhibiting biofilm formation by clinical isolates with diverse matrix components. Moreover, NGM affected multiple phenotypes in addition to biofilm formation, including cell morphology and β-lactam susceptibility. Notably, compared with NGM, 17DN exerted a moderate effect on these phenotypes, suggesting that the acetyl group of NGM is recognized by common factor(s) controlling these phenotypes. The target molecule of NGM is not yet known. Understanding the mechanism of action of NGM will provide insights that will facilitate the development of drugs to prevent and cure staphylococcal biofilm infections.

## Methods

### Bacterial strains

*S. aureus* and *S. epidermidis* strains (Table S1) were cultured in BHI broth (Becton Dickinson, Franklin Lakes, NJ, USA) at 37 °C.

### Biofilm formation

Overnight cultures of *S*. *aureus* and *S*. *epidermidis* grown in BHI broth were diluted 500-fold in BHIG broth, 5% DMSO, with or without 50 μM of test compounds unless otherwise noted. Aliquots of the cell suspensions (200 µL) were incubated in 96-well flat-bottomed polystyrenes plates (Corning, Corning, NY, USA) at 37 °C for 24 h. Bacterial growth was assessed by measuring the absorbance of cultures at 595 nm using a microplate reader (Infinite 200 PRO; Tecan, Mannedorf, Switzerland). After removing culture supernatants and planktonic cells, biofilms that formed on the bottoms of the wells were washed twice with 200 µL of phosphate-buffered saline (PBS), and the adherent biofilms were stained using 100 µL of 0.5% crystal violet for 1 min and washed twice with 200 μL of PBS. Finally, biofilm formation was quantified by measuring absorbance at 595 nm using a microplate reader (Infinite 200 PRO).

### Half maximal (50%) inhibitory concentration (IC_50_)

The IC_50_ was calculated using the following formula: IC_50_ = 10^[Log (A/B)^^×^^(50^ ^− C)/(D^^−^^C)^^+^^Log (B)]^. A: Corresponding concentrations of test compound directly above 50% inhibition, B: Corresponding concentrations of test compound directly below 50% inhibition, C: % inhibition directly below 50% inhibition and D: % inhibition directly above 50% inhibition.

### High-throughput screening

To isolate compounds that inhibit biofilm formation in *S*. *aureus* MR23, we conducted high-throughput screening of 50,000 compounds stocked at the Drug Discovery Initiative of The University of Tokyo. Quantification of biofilm formation was conducted automatically using a custom-built FreedomEVO System (Tecan). The final concentration of each compound was 20 μM.

### Biofilm inhibitors

The biofilm inhibitors used in this study were purchased from the following companies: NGM (Sigma-Aldrich, St. Louis, MO, USA); 17DN (Toronto Research Chemicals, Toronto, Canada); MBX-1246, MBX-1240, and ABC-1 (Vitas-M Laboratory, Ltd., Hong Kong, China); MBX-1384 (Enamine Ltd., Kiev, Ukraine); D-tyrosine (Sigma-Aldrich) and tannic acid (Wako Pure Chemical Industries, Ltd.).

### Analysis of bacterial growth

An overnight culture of *S. aureus* MR23 was diluted 500-fold into fresh BHI broth with or without 50 μM of test compound, and 200-μL aliquots were cultured in 96-well flat -bottomed polystyrene plates (Corning) at 37 °C for 24 h with shaking. The absorbance of each culture at 600 nm was measured every 30 min for 24 h using a Bio Microplate Reader HiTS (Scinics Corp., Tokyo, Japan).

### Fluorescent labeling of extracellular PIA

Staphylococci were stained using WGA-Alexa 488 (Invitrogen, Carlsbad, CA, USA) according to a previously published method, with minor modifications.^45^ *S. aureus* and *S. epidermidis* were cultured using the biofilm conditions detailed above, except that 2-mL aliquots were cultured in 12-well flat-bottomed polystyrenes plates (Corning). After 24 h of cultivation, if required, 20 μg/mL of DspB (Kane Biotech Inc., Winnipeg, Canada) was added to the biofilm, and the mixture was incubated for 2 h at 37 °C. Then, the cells were washed, suspended in PBS, stained with 5 μg/mL of WGA-Alexa 488 in PBS for 20 min at 37 °C, washed twice with PBS, and observed using a fluorescence microscope (ECLIPSE E600; Nikon, Tokyo, Japan).

### ECM extraction

ECMs were extracted based on previously described methods.^45^ Biofilm cells, cultured for 24 h in the presence or absence of 50 μM of the test compound, were harvested by centrifugation at 5000 × *g* and 25 °C for 10 min. After washing with PBS, 100 mg of the cells was suspended in 1000 μL of extraction buffer (Tris-HCl pH 8.0; 1.5 M NaCl), and incubated at 25 °C for 30 min with agitation. Then, cells were removed by centrifugation at 15,000 × *g* and 25 °C for 10 min, and extracted ECMs in the supernatants were stored at −20 °C until use.

### Dot blot analysis

ECMs extracted from cells of *S*. *aureus* SH1000 and MR23 were diluted 200-fold in PBS. Those extracted from cells of *S*. *epidermidis* SE4 were diluted 1600-fold. A nitrocellulose membrane (Bio-Rad, Hercules, CA, USA) was placed in the Bio-Dot apparatus (Bio-Rad), according to the manufacturer’s instructions. Two hundred microliters of the diluted ECMs were spotted onto the membrane. After vacuuming, the membrane was blocked using blocking buffer consisting of 0.5% skim milk in Tris-buffered saline with 0.05% Tween 20 (TBS-T) for 30 min at 25 °C. Then, the membrane was incubated with 0.1 µg/mL WGA-horseradish peroxidase (HRP; J-OIL Mills, Inc., Tokyo, Japan) in the blocking buffer for 30 min. After washing three times with TBS-T, PIA signal was visualized using ECL prime (GE Healthcare, Chalfont St. Giles, UK).

### Quantification of ECM proteins

The amount of proteins in the ECM was measured using a Qubit 3.0 Fluorometer (Thermo Fisher Scientific, Waltham, MA, USA) according to the manufacturer’s instructions.

### 2-D electrophoresis

*S. aureus* was cultured were cultured for 24 h in the presence or absence of 50 μM of the test compound under biofilm-forming conditions and harvested from 50 mL of culture medium by centrifugation at 5000 × *g* for 5 min at 4 °C. Cell pellets were suspended in 1 mL of PBS buffer containing 0.2 mM dithiothreitol (DTT), cOmplete Mini, EDTA-free protease inhibitor cocktail (Roche). The cell suspensions were centrifuged at 5000×*g* for 10 min at 4 °C and resuspended in 1 mL of lysis buffer (0.2 mg/mL lysostaphin; 10 mM Tris-HCl pH 8.0; 0.2 mM DTT; DNase I; RNase A and cOmplete Mini, EDTA-free protease inhibitor cocktail). After incubation for 30 min at 37 °C, cells were ultrasonically disrupted using a Branson Sonifier 250AA/450AA (Branson Ultrasonics Corp., Danbury, CT, USA). Cell lysates were centrifuged at 15,000 × *g* for 10 min at 4 °C, and the supernatants were collected. Prior to 2-D electrophoresis, protein concentrations were determined, and samples were prepared using a 2-D Quant Kit and a 2-D Clean-Up Kit (GE Healthcare), respectively, according to the manufacturer’s instructions. The proteins (100 µg) were subjected to isoelectric focusing using a WSE-1500 Disc Gel EP Kit (ATTO Corp., Tokyo, Japan) with Agar GEL pH 4–6 (ATTO). Second-dimension vertical SDS-PAGE was performed using a WSE-1150M pageRunAce (ATTO).

### Cytoplasmic protein extraction

Biofilm cells, cultured for 24 h in the presence or absence of 50 μM of the test compound, were harvested by centrifugation at 5000×*g* and 25 °C for 10 min and washed once with PBS. Then, pellets were resuspended in PBS containing cOmplete Mini EDTA-free protease inhibitor cocktail (Roche, Indianapolis, IN, USA) and 0.2 mg/mL of lysostaphin (Wako Pure Chemical Industries, Ltd., Osaka, Japan). Cells were lysed by sonication, and the supernatant was collected after centrifugation (15,000×*g* and 4 °C for 10 min). The protein concentrations were determined using a Pierce BCA Protein Assay Kit (Thermo Fisher Scientific).

### Western blotting analysis

ECMs (5 μL) or cytoplasmic proteins (1 μg) were subjected to SDS-PAGE, and then electroblotted onto a nitrocellulose membrane (ATTO). Subsequently, the membrane was blocked using blocking buffer consisting of 5% skim milk in PBS with 0.05% Tween 20 (PBS-T) for 2 h at 25 °C. After incubation with a primary antibody followed by a secondary antibody, signals derived from enolase were visualized using ECL prime (GE Healthcare). For detection of enolase, the primary (anti-enolase polyclonal antibody) and secondary antibodies (HRP-conjugated anti-rabbit IgG antibody, Bio-Rad) were diluted 1:10,000 and 1:10,000 in the blocking buffer, respectively. For detection of PBP2a, the primary (mouse anti-PBP2a antibody, product code: 130-10096-200, Raybiotech, Inc., Norcross, GA, USA) and secondary antibodies (HRP-conjugated anti-mouse IgG antibody, Bio-Rad) were diluted 1:1000 and 1:10,000 in the blocking buffer, respectively.

### Transmission electron microscopy (TEM)

*S. aureus* MR23 and SH1000 were cultured under biofilm conditions in the presence or absence of 50 μM of the test compound, and the cells were harvested by centrifugation at 5000×*g* for 10 min and fixed with 2.5% glutaraldehyde. The fixed samples were observed using a TEM H-7500 (Hitachi, Ltd., Tokyo, Japan), and images were captured. The cell wall thicknesses of 50 randomly selected cells were measured. The number of cells with abnormal septal formation in 150 randomly selected cells, in which septal formation was observed along the equator of the cell, was counted.

### RNA extraction

*S. aureus* MR23 was grown under biofilm conditions for 12 h in the presence or absence of 50 μM of the test compound. Cells were harvested by centrifugation at 5000×*g* at 25 °C for 10 min and immediately incubated with an appropriate volume of RNA Protect (Qiagen, Hilden, Germany) for 5 min. Cells were collected by centrifugation at 5000×*g* at 25 °C for 10 min and stored at −80 °C. Total RNA was extracted using an RNeasy Maxi Kit (Qiagen) according to the manufacturer’s instructions, except that cells were treated with 0.2 mg/mL lysostaphin at 37 °C for 30 min before extraction.

### Microarray analysis

The effect of each compound on the global abundance of gene transcripts was analyzed using a GeneChip *S. aureus* Genome Array (Affymetrix, Santa Clara, CA, USA). A cDNA synthesis kit (ReverTra Ace qPCR RT Kit; TOYOBO, Osaka, Japan) was used to immediately convert the RNA to cDNA to avoid RNA degradation. Hybridization, washing, and scanning of cDNA on the array were performed according to the manufacturer’s instructions. The arrays were analyzed using GeneChip software, and global scaling was used to normalize the data acquired from different arrays. Spotfire DecisionSite 9.1.1 was used to analyze the data. The datasets represented cultures treated with DMSO and NGM (two each) (GEO Accession: GSE90857).

### Quantitative real-time PCR

Primers were designed according to the published sequences of *S. aureus* genes, using Primer Express 3.0.1 software (Applied Biosystems, Foster City, CA, USA). The primers used in this study are listed in Table S9. Real-time PCR was performed using a Step One plus (Applied Biosystems) and SYBR green PCR master mix (Applied Biosystems). Reactions (20 μL per sample) were performed in triplicate using 96-well plates. All reactions contained 0.1 μg of cDNA, 10 μL of SYBR Green Master Mix, 1 μL of each primer and sterile water that had been treated twice with RNase. RNA levels of the 16 S ribosomal RNA gene were used to normalize transcript levels. Amplification was performed using a Step One plus (Applied Biosystems) using the following program: 10 min at 95 °C, 35 cycles for 15 s at 95 °C, and 60 s at 60 °C. After PCR, melting curve analysis was performed as follows: 15 s at 95 °C, 60 s at 60 °C, and 15 s at 95 °C. Relative gene expression levels were calculated according to the differences in cycle thresholds (ΔCT) of all samples. PCR experiments were performed in triplicate.

### Localization and analysis of PBPs using fluorescent penicillin

MR23 cells were mixed with fluorescent penicillin (BOCILLIN FL, Thermo Fisher Scientific) at a final concentration of 10 μM for 30 min at 37 °C, washed twice with PBS, and suspended in PBS. Cells were observed using a fluorescence microscope. Fluorescence intensities of the suspensions were measured with an Infinite 200 PRO microplate reader at excitation and emission wavelengths of 485 and 535 nm, respectively.

### Fluorescence detection of PBP2

Cytoplasmic proteins (5 µg) and 10 μM of BOCILLIN FL (Thermo Fisher Scientific) were incubated at 37 °C for 30 min, denatured with SDS-denaturing solution at 100 °C for 3 min, and each reaction mixture was subjected to SDS-PAGE analysis, as described previously.^46^ To visualize labeled PBP2, the gels were scanned under blue light using an Image Quant LAS 4000 (GE Healthcare).

### Antibiotic susceptibility testing

The MIC was determined using a Dry Plate Eiken (Eiken Chemical Co., Tokyo, Japan) according to the manufacturer’s protocol. When the MIC was outside the measuring range of the Dry Plate Eiken, it was determined using a laboratory-made plate containing appropriate amounts of antimicrobial agents. Antibiotic susceptibility testing was performed according to the standardized broth microdilution method recommended by the Clinical Laboratory Standards Institute. Briefly, approximately 5 × 10^4^ colony-forming units of *S. aureus* were inoculated into 100 μL of Mueller-Hinton or BHIG broth containing 5% DMSO, in the presence or absence of 50 μM of the test compound. The antimicrobial agents used in this study are listed in Table 2 and Tables S5–S8. The lowest concentration that visibly inhibited bacterial growth was defined as the MIC.

### Data availability

All data except for microarray data that support the findings of this study are available in the present manuscript in either the main text or the supplementary information. The datasets generated by microarray are available in the Gene Expression Omnibus repository (GEO Accession: GSE90857), https://www.ncbi.nlm.nih.gov/geo/query/acc.cgi?acc=GSE90857.

## Electronic supplementary material

Supplementary information

Table S1. Strains and plasmids used in this study

Table S2. Biofilm inhibitory activities of compounds previously reported against various staphylococcal strains

Table S3. Microarray and real-time PCR of sasG and the gene encoding enolase, eno

Table S4. Microarray and real-time PCR of peptidoglycan synthetases and hydrolases

Table S5. The effects of NGM on antibiotic susceptibility of MRSA strains in Muller-Hinton broth

Table S6. The effects of NGM on antibiotic susceptibility of MRSA strains in BHIG broth

Table S7. The effects of NGM on antibiotic susceptibility of MSSA strains in Mueller-Hinton broth

Table S8. The effects of NGM on antibiotic susceptibility of MSSA strains in BHIG broth

Table S9. Primers for real-time PCR used in this study

Figure S1. Fluorescence microscopy of PIA

Figure S2. Quantification of PIA by dot blot assay

Figure S3. The effects of NGM and 17DN on cell morphology

Figure S4. Expression levels of PBP2 and PBP2a with total protein stains as loading controls
