# Author Correction: Norgestimate inhibits staphylococcal biofilm formation and resensitizes methicillin-resistant *Staphylococcus aureus* to β-lactam antibiotics

**DOI:** 10.1038/s41522-017-0038-x

**Published:** 2017-11-08

**Authors:** Yutaka Yoshii, Ken-ichi Okuda, Satomi Yamada, Mari Nagakura, Shinya Sugimoto, Tetsuo Nagano, Takayoshi Okabe, Hirotatsu Kojima, Takeo Iwamoto, Kazuyoshi Kuwano, Yoshimitsu Mizunoe

**Affiliations:** 1 Department of Bacteriology, The Jikei University School of Medicine, 3-25-8 Nishi-Shimbashi, Minato-ku, Tokyo, 105-8461 Japan; 2 Jikei Center for Biofilm Science and Technology, 3-25-8 Nishi-Shimbashi, Minato-ku, Tokyo, 105-8461 Japan; 3 Division of Respiratory Diseases, Department of Internal Medicine, The Jikei University School of Medicine, 3-25-8 Nishi-Shimbashi, Minato-ku, Tokyo, 105-8461 Japan; 4 Drug Discovery Initiative, The University of Tokyo, 7-3-1 Hongo, Bunkyo-ku, Tokyo, 113-0033 Japan; 5 Division of Molecular Cell Biology, Core Research Facilities for Basic Science, The Jikei University School of Medicine, 3-25-8 Nishi-Shimbashi, Minato-ku, Tokyo, 105-8461 Japan

**Correction to:** *npj Biofilms and Microbiomes* (2017); doi:10.1038/s41522-017-0026-1; Published 21 July 2017

At proofing a reference was removed, but references in Fig. 2, Supplementary Information, Supplementary Table 1, and Supplementary Table 9 were not updated to reflect the change. These citations have now been corrected in the revised Figure and Supplementary Information files, in the HTML and PDF versions of this article. A correction has been published and is appended to both the HTML and PDF versions of this paper. The error has been fixed in the paper.

**Fig. 2:**
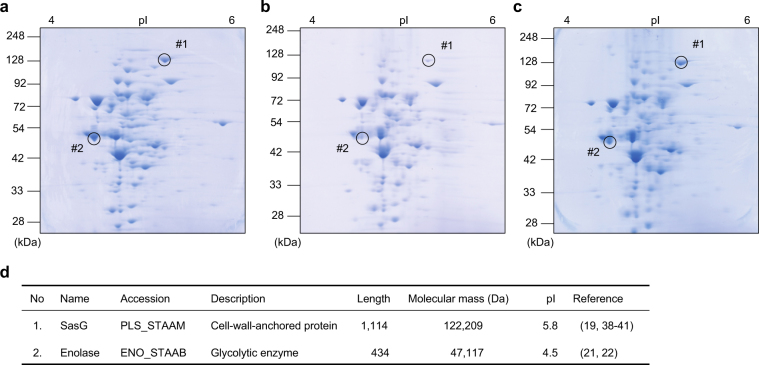
The effects of NGM on the proteome of *S. aureus*. **a**–**c** Two-dimensional (2-D) electrophoresis in the presence or absence of NGM or 17DN (**a** DMSO control; **b** NGM; **c** 17DN). **d** Identification of proteins, whose expressions were decreased in the presence of NGM on 2-D gel

