# Supplementary material for: Norgestimate inhibits staphylococcal biofilm formation and resensitizes methicillin-resistant Staphylococcus aureus to β-lactam antibiotics
Source: NPJ Biofilms Microbiomes. 2017 Jul 21;3:18. doi: 10.1038/s41522-017-0026-1 (PMC5522392; doi:10.1038/s41522-017-0026-1)
Supplement: Supplementary file 1 — Supplementary information [file 41522_2017_26_MOESM1_ESM.docx]

**SUPPLEMENTARY INFORMATION**

**Supplementary Methods**

*In-gel digestion*

Spots with markedly decreased intensities, corresponding to proteins isolated from cells treated with 50 μM of NGM, were excised from the gels and stained with Coomassie Brilliant Blue, destained twice using 100 μL of 25 mM ammonium bicarbonate in 50% acetonitrile for 10 min and 100 μL of 25 mM ammonium bicarbonate in 30% acetonitrile for 10 min. After discarding the washing solution, the gel pieces were dehydrated with 200 μL of 100% acetonitrile and vacuum-dried. The disulfide bonds in the proteins in the gels were reduced with 10 mM DTT in 25 mM ammonium bicarbonate at 56°C for 45 min, washed once with 200 μL of 25 mM ammonium bicarbonate and alkylated with 10 mg/mL of iodoacetamide in 25 mM ammonium bicarbonate at 37°C for 30 min. The gel pieces were washed once with 200 μL of 25 mM ammonium bicarbonate and dehydrated with 100 μL of 25 mM ammonium bicarbonate in 50% acetonitrile followed by 200 μL of 100% acetonitrile. The proteins in the vacuum-dried gels were digested using 20 μL of 20 ng/μL Trypsin/Lys-C Mix (Promega, Madison, WI, USA) at 37°C overnight. The peptides were extracted from the gels using 50 μL of 50% acetonitrile containing 5% trifluoroacetic acid at 25°C for 30 min. After removing the acetonitrile by evaporation, the peptides were analyzed using nano ultra-high-pressure liquid chromatography (UHPLC)-MS/MS.

*Nano-UHPLC and Electrospray Ionization Tandem Mass Spectrometry (ESI-MS/MS)*

Peptides were desalted by injected them into a Magic C18 AQ reverse-phase UHPLC nano trap column (5 μm, 200 Å; Bruker Daltronics, Bremen, Germany). Peptides were separated using a C18 reversed-phase Nano column (100 μm ID × 30 cm, Zaplous αX PepC18, 3 μm, 120 Å; AMR, Tokyo, Japan) using a nano-flow linear gradient (acetonitrile, 0.1% formic acid). Nano UHPLC was performed automatically using a trap column-switching device coupled to an auto-sampler and a nano-gradient generator (nano-Advance; Michrom Bioresources, Inc., Auburn, CA, USA). Hystar 3.2-SR software was used to control the entire process. The eluted peptides were delivered to an ultra-high resolution electrospray ionization quadrupole time-of-ﬂight mass spectrometer (Maxis3G, Bruker Daltronics) equipped with a CaptiveSpray source (Bruker Daltronics). The mass spectrometer was operated in data-dependent MS/MS mode to alternatively acquire full scans (*m/z* 100–2200 Da/e). The four most intense peaks in any full scan were selected as precursor ions and fragmented using collision energy. MS/MS spectra were interpreted and peak lists were generated using Compass DataAnalysis 4.1 and Biotools 3.2 software (Bruker Daltronics).

*Bioinformatics*

Peptide masses were compared with those included in NCBInr.2016.02.20 using an in-house licensed MASCOT server (Matrix Science Ltd., London, England). The parameters used for all searches were as follows: maximum number of missed cleavages allowed = 1, the mass tolerance = 0.5 Da, minimum of four peptides required to match. Mono-isotopic masses were used to match the calculated mono-isotopic fragment mass for protein identification.

*Protein expression and purification*

The gene encoding *S*. *aureus* enolase was amplified from the genome of *S*. *aureus* MR23 by PCR using the following set of primers: eno_Forward (atgccaattattacagatgtttacgc) and eno_Reverse (ttatttatctaagttatagaatgatttgataccgtc). The amplified PCR fragment was cloned into pCold I vector (Takara Bio, Otsu, Japan; Table S1) using In-Fusion HD Cloning Kit (Takara Bio) according to the manufacturer’s protocol. The resulting plasmid pCold_*eno* was amplified in *Escherichia* *coli* DH5α, and subsequently introduced into *E*. *coli* BL21(DE3) by electroporation. *E*. *coli* BL21(DE3) harboring pCold_*eno* was cultured in LB broth containing 100 μg/mL ampicillin at 37°C. When the OD_600_ reached 0.5, the culture was cooled and kept at 15°C for 30 min. Then, isopropyl β-D-1-thiogalactopyranoside was added to a final concentration of 0.5 mM to induce expression. After cultivation for 24 h at 15°C, the cells were collected by centrifugation and resuspended in 20 mM Hepes-NaOH (pH 7.4), 150 mM NaCl (buffer A) containing cOmplete Mini, EDTA-free protease inhibitor cocktail (Roche). The collected cells were disrupted by ultrasonication and insoluble materials were removed by two rounds of centrifugation at 8,000 × *g* for 10 min. To purify hexa-histidine tagged enolase (His_6_-enolase), TALON Metal Affinity Resins (Clontech Laboratories, Palo Alto, CA) were added to the recovered supernatant and incubated for 2 h at 4°C with rotation. After washing the resin with buffer A containing 5 mM imidazole, the protein was eluted with buffer A containing 50 mM imidazole. The eluted His_6_-enolase was further subjected to size exclusion chromatography using a Superdex 75 10/300 column (GE Healthcare) equilibrated in buffer A containing 10% glycerol. Fractions containing His_6_-enolase were pooled and stored at −80°C until use.

*Antibody production and purification*

An anti-enolase polyclonal antibody was generated in rabbits using the purified enolase as an antigen. The serum was collected and the polyclonal antibodies were purified using a HiTrap protein G column (GE Healthcare) according to the manufacturer’s protocol. The purified antibody was stored at 4°C until use.

**SUPPLEMENTAL REFERENCES**

**47.** Horsburgh, M. J. *et al.* σ^B^ modulates virulence determinant expression and stress resistance: characterization of a functional *rsbU* strain derived from *Staphylococcus aureus* 8325-4. *J. Bacteriol.* **184,** 5457–67 (2002).

**48.** Smith, K. *et al.* Influence of tigecycline on expression of virulence factors in biofilm-associated cells of methicillin-resistant *Staphylococcus aureus*. *Antimicrob. Agents Chemother.* **54,** 380–7 (2010).

**49.** Levinger, O., Bikels-Goshen, T., Landau, E., Fichman, M. & Shapira, R. Epigallocatechin gallate induces upregulation of the two-component VraSR system by evoking a cell wall stress response in *Staphylococcus aureus*. *Appl. Environ. Microbiol.* **78,** 7954–9 (2012).

**SUPPLEMENTARY FIGURE LEGENDS**

**Figure S1. Fluorescence microscopy of PIA.** PIA and DNA were stained using WGA (upper panels) and DAPI (lower panels), respectively. Arrowheads indicate filamentous structural PIA. Scale bars = 5 μm. In these experiments, cells were cultured under biofilm-forming conditions, with or without 50 μM of the test compound, or 20 μg/mL of DspB. Abbreviations: Cont., control; NGM, norgestimate; 17DN, 17-deacetylnorgestimate; DspB, dispersin B

**Figure S2. Quantification of PIA by dot blot assay.** (a) SH1000; (b) MR23; (c) SE4. Data are presented as the mean ± SD (standard deviation) (*n* = 3). **p* < 0.05, ***p* < 0.01. In these experiments, cells were cultured under biofilm-forming conditions, with or without 50 μM of the test compound, or 20 μg/mL of DspB. Abbreviations: Cont., control; NGM, norgestimate; 17DN, 17-deacetylnorgestimate; DspB, dispersin B

**Figure S3. The effects of NGM and 17DN on cell morphology.** (a) TEM images. Yellow arrowheads indicate abnormal septal formation, and white arrowheads indicate normal septal formation in each cell. Scale bars = 1 μm. (b) Cell wall thickness of MR23 cells cultured in the presence or absence of NGM at 6, 12, and 24 h. Data are presented as the mean ± SD (*n* = 50) **p* < 0.01. In these experiments, cells were cultured under biofilm-forming conditions, with or without 50 μM of the test compound. Abbreviations: Cont., control; NGM, norgestimate; 17DN, 17-deacetylnorgestimate

**Figure S4. Expression levels of PBP2 and PBP2a with total protein stains as loading controls.** (a) CBB staining of proteins by PBP2 detection analysis. (b–d) CBB staining of proteins by Western blotting analysis of PBP2a (b, DMSO control; c, NGM; d, 17DN). In these experiments, cells were cultured under biofilm-forming conditions, with or without 50 μM of the test compound. Abbreviations: Cont., control; NGM, norgestimate; 17DN, 17-deacetylnorgestimate
