# Supplementary material for: Norgestimate inhibits staphylococcal biofilm formation and resensitizes methicillin-resistant Staphylococcus aureus to β-lactam antibiotics
Source: NPJ Biofilms Microbiomes. 2017 Jul 21;3:18. doi: 10.1038/s41522-017-0026-1 (PMC5522392; doi:10.1038/s41522-017-0026-1)
Supplement: Supplementary file 2 — Table S1. Strains and plasmids used in this study [file 41522_2017_26_MOESM2_ESM.docx]

Table S1. Strains and plasmids used in this study

| Strain | Description | Source or reference |
| --- | --- | --- |
| *Staphylococcus aureus* strain |  |  |
| MS3 | MSSA strain isolated in the Jikei Hospital | Sugimoto, *et al.*, unpublished data |
| MS4-5 | MSSA strain isolated in the Jikei Hospital | Sugimoto, *et al.*, unpublished data |
| MS18 | MSSA strain isolated in the Jikei Hospital | Sugimoto, *et al.*, unpublished data |
| SH1000 | *S. aureus* strain 8325-4 with functional *rsbU* | (47) |
| MR2 | MRSA strain isolated in the Jikei Hospital | Sugimoto, *et al.*, unpublished data |
| MR4 | MRSA strain isolated in the Jikei Hospital | Sugimoto, *et al.*, unpublished data |
| MR11 | MRSA strain isolated in the Jikei Hospital | Sugimoto, *et al.*, unpublished data |
| MR23 | MRSA strain isolated in the Jikei Hospital | (17, 45) |
| *Staphylococcus epidermidis* strain |  |  |
| SE4 | *S. epidermidis* strain isolated in the Jikei Hospital | (45) |
| SE21 | *S. epidermidis* strain isolated in the Jikei Hospital | Sugimoto, *et al.*, unpublished data |
| *Escherichia* *coli* strain |  |  |
| DH5α | deoR, endA1, gyrA96, hsdR17(r_k_^-^, m_k_^+^), phoA, recA1, relA1, *sup*E44, *thi*-1, Δ(lacZYA-argF)U169, f80dlacZΔM15, F^-^, λ^-^ | Toyobo |
| BL21(DE3) | F–, *omp*T, *hsd*S_B_ (r_B_^–^, m_B_^–^), *dcm*, *gal*, λ(DE3), pLysS, Cm^r^ (λ(DE3): *lac*I, *lac*UV5-T7 gene 1, *ind*1, *sam*7, *nin*5) | Promega |
| Plasmids |  |  |
| pCold I | An cold-shock expression vector | Takara Bio |
| pCold_*eno* | *eno* cloned in pCold I under the control of a cold-shock promoter | in this study |
