# Supplementary material for: Norgestimate inhibits staphylococcal biofilm formation and resensitizes methicillin-resistant Staphylococcus aureus to β-lactam antibiotics
Source: NPJ Biofilms Microbiomes. 2017 Jul 21;3:18. doi: 10.1038/s41522-017-0026-1 (PMC5522392; doi:10.1038/s41522-017-0026-1)
Supplement: Supplementary file 3 — Table S2. Biofilm inhibitory activities of compounds previously reported against various staphylococcal strains [file 41522_2017_26_MOESM3_ESM.docx]

Table S2. Biofilm inhibitory activities of compounds previously reported against various staphylococcal strains

| Strain | IC_50_^*^ value (μM) | | | | | | | |
| --- | --- | --- | --- | --- | --- | --- | --- | --- |
|  | NGM^†^ | 17DN^‡^ | MBX-1240^§^ | MBX-1246^§^ | MBX-1384^§^ | ABC-1^¶^ | D-tyrosine^#^ | Tannic acid^\|\|^ |
| MSSA |  |  |  |  |  |  |  |  |
| MS3 | 22.5 | 92.2 | 96.3 | 27.3 | 27.2 | >100 | >100 | >100 |
| MS4-5 | 22.4 | 78.5 | 32.2 | 11.9 | 14.9 | >100 | >100 | >100 |
| MS18 | 13.1 | 75.6 | 46.0 | 15.7 | 21.5 | >100 | >100 | >100 |
| SH1000 | 15.6 | 49.0 | 47.3 | 14.7 | 16.7 | >100 | >100 | >100 |
| MRSA |  |  |  |  |  |  |  |  |
| MR2 | 13.4 | 76.5 | 46.6 | 21.8 | 27.2 | >100 | >100 | >100 |
| MR4 | 13.8 | 84.0 | 27.3 | 12.4 | 16.0 | >100 | >100 | >100 |
| MR11 | 12.0 | 62.7 | 37.1 | 14.0 | 14.4 | >100 | >100 | >100 |
| MR23 | 15.9 | 95.6 | 26.5 | 13.3 | 13.4 | >100 | >100 | >100 |
| *Staphylococcus epidermidis* |  |  |  |  |  |  |  |  |
| SE4 | 15.1 | >100 | >100 | 64.4 | >100 | >100 | >100 | >100 |
| SE21 | 14.6 | >100 | >100 | 47.6 | >100 | >100 | >100 | >100 |

^*^Half maximal inhibitory concentration. ^†^Norgestimate. ^‡^17-deacetyl norgestimate. ^§^These compounds were referenced from (12).

^¶^ABC-1 was referenced from (13). ^#^D-tyrosine was referenced from (14). ^||^Tannic acid was referenced from (15).
