# Supplementary material for: Norgestimate inhibits staphylococcal biofilm formation and resensitizes methicillin-resistant Staphylococcus aureus to β-lactam antibiotics
Source: NPJ Biofilms Microbiomes. 2017 Jul 21;3:18. doi: 10.1038/s41522-017-0026-1 (PMC5522392; doi:10.1038/s41522-017-0026-1)
Supplement: Supplementary file 4 — Table S3. Microarray and real-time PCR of sasG and the gene encoding enolase, eno [file 41522_2017_26_MOESM4_ESM.docx]

Table S3. Microarray and real-time PCR of *sasG* and the gene encoding enolase, *eno*

| No | Gene name | Description | Microarray |  | Real-time PCR | | |
| --- | --- | --- | --- | --- | --- | --- | --- |
|  |  |  | NGM/Cont. |  | NGM/Cont. | NGM/17DN | 17DN/Cont. |
|  |  |  | (Fold change) |  | (Fold change) | (Fold change) | (Fold change) |
| 1. | *sasG* | cell wall-anchored surface protein G | 0.40 |  | 2.73 | 1.51 | 1.81 |
| 2. | *eno* | enolase (2-phosphoglycerate dehydrogenase) | 0.56 |  | 0.36 | 0.64 | 0.57 |
