# Supplementary material for: Norgestimate inhibits staphylococcal biofilm formation and resensitizes methicillin-resistant Staphylococcus aureus to β-lactam antibiotics
Source: NPJ Biofilms Microbiomes. 2017 Jul 21;3:18. doi: 10.1038/s41522-017-0026-1 (PMC5522392; doi:10.1038/s41522-017-0026-1)
Supplement: Supplementary file 5 — Table S4. Microarray and real-time PCR of peptidoglycan synthetases and hydrolases [file 41522_2017_26_MOESM5_ESM.docx]

Table S4. Microarray and real-time PCR of peptidoglycan synthetases and hydrolases

| Gene name | Description | Microarray |  | Real-time PCR | | | | |
| --- | --- | --- | --- | --- | --- | --- | --- | --- |
|  |  | NGM/Cont. |  | NGM/Cont. |  | NGM/17DN |  | 17DN/Cont. |
|  |  | (Fold change) |  | (Fold change) |  | (Fold change) |  | (Fold change) |
| Peptidoglycan synthetases | |  |  |  |  |  |  |  |
| *murA* | UDP-N-acetylglucosamine 1-carboxyvinyltransferase | 0.72 |  | —^*^ |  | —^*^ |  | —^*^ |
| *murB* | UDP-N-acetylenolpyruvoylglucosamine reductase | 1.21 |  | —^*^ |  | —^*^ |  | —^*^ |
| *murC* | UDP-N-acetylmuramate--L-alanine ligase | 1.09 |  | —^*^ |  | —^*^ |  | —^*^ |
| *murE* | UDP-N-acetylmuramoyl-L-alanyl-D-glutamate--2, 6-diaminopimelate ligase | 1.64 |  | —^*^ |  | —^*^ |  | —^*^ |
| *mraY* | phospho-N-acetylmuramoyl-pentapeptide-transferase | 1.26 |  | —^*^ |  | —^*^ |  | —^*^ |
| *murG* | N-acetylglucosaminyl transferase | 1.32 |  | —^*^ |  | —^*^ |  | —^*^ |
| *femA* | glycine interpeptide bridge formation protein FemA | 3.58 |  | 2.09 |  | 1.36 |  | 1.53 |
| *femC* | glutamine synthetase FemC | 2.64 |  | 8.56 |  | 3.08 |  | 2.78 |
| *pbp1* | penicillin-binding protein 1 | 1.28 |  | 2.14 |  | 1.38 |  | 1.54 |
| *pbp2* | penicillin-binding protein 2 | 2.68 |  | 2.38 |  | 1.68 |  | 1.42 |
| *pbp3* | penicillin-binding protein 3 | 1.75 |  | 1.80 |  | 1.11 |  | 1.62 |
| *pbp4* | penicillin-binding protein 4 | 1.34 |  | 3.66 |  | 2.12 |  | 1.72 |
| Peptidoglycan hydrolases | |  |  |  |  |  |  |  |
| *atl* | bifunctional N-acetylmuramoyl-L-alanine amidase/endo-beta-N-acetylglucosaminidase | 3.17 |  | 5.91 |  | 3.87 |  | 1.52 |
| *isaA* | immunodominant antigen A, transglycosylase IsaA | 5.21 |  | 4.66 |  | 2.79 |  | 1.67 |
| *lytM* | glycyl-glycine endopeptidase lytM | 5.31 |  | 12.91 |  | 6.24 |  | 2.07 |

*Not analyzed.
