# Supplementary material for: Norgestimate inhibits staphylococcal biofilm formation and resensitizes methicillin-resistant Staphylococcus aureus to β-lactam antibiotics
Source: NPJ Biofilms Microbiomes. 2017 Jul 21;3:18. doi: 10.1038/s41522-017-0026-1 (PMC5522392; doi:10.1038/s41522-017-0026-1)
Supplement: Supplementary file 8 — Table S7. The effects of NGM on antibiotic susceptibility of MSSA strains in Mueller-Hinton broth [file 41522_2017_26_MOESM8_ESM.docx]

Table S7. The effects of NGM on antibiotic susceptibility of MSSA strains in Mueller-Hinton broth

| Antibiotic | MIC^*^ (μg/mL) | | | | | | | | | | | | | | |
| --- | --- | --- | --- | --- | --- | --- | --- | --- | --- | --- | --- | --- | --- | --- | --- |
|  | MS3 | | |  | MS4-5 | | |  | MS18 | | |  | SH1000 | | |
|  | Control | NGM^†^ | 17DN^‡^ |  | Control | NGM | 17DN |  | Control | NGM | 17DN |  | Control | NGM | 17DN |
| **β-lactam** |  |  |  |  |  |  |  |  |  |  |  |  |  |  |  |
| Oxacillin | 0.25 | 0.06 | 0.25 |  | 0.5 | 0.12 | 0.25 |  | 0.25 | 0.06 | 0.12 |  | 0.5 | 0.12 | 0.25 |
| Ampicillin | 4 | 0.25 | 0.5 |  | 8 | 0.5 | 4 |  | 4 | 0.12 | 0.25 |  | 4 | 0.12 | 0.12 |
| Cefazolin | 1 | 0.5 | 0.5 |  | 1 | 0.25 | 0.5 |  | 1 | 0.12 | 0.5 |  | 1 | 0.5 | 0.5 |
| Cefmetazole | 2 | 1 | 1 |  | 2 | 0.25 | 0.5 |  | 2 | 0.5 | 1 |  | 2 | 0.5 | 1 |
| Flomoxef | 1 | 0.25 | 0.5 |  | 0.5 | 0.12 | 0.5 |  | 0.5 | 0.25 | 0.5 |  | 0.5 | 0.25 | 0.5 |
| Cefoxitin | 4 | 2 | 4 |  | 4 | 2 | 4 |  | 4 | 2 | 4 |  | 4 | 2 | 4 |
| Imipenem | 0.5 | 0.25 | 0.25 |  | 0.25 | 0.06 | 0.12 |  | 0.25 | 0.12 | 0.12 |  | 0.25 | 0.12 | 0.25 |
| **Glycopeptide** |  |  |  |  |  |  |  |  |  |  |  |  |  |  |  |
| Vancomycin | 2 | 1 | 1 |  | 1 | 0.5 | 0.5 |  | 1 | 0.5 | 1 |  | 2 | 1 | 1 |
| Teicoplanin | 2 | 1 | 1 |  | 0.5 | 0.5 | 0.5 |  | 1 | 0.5 | 1 |  | 1 | 1 | 0.5 |
| **Oxazolidinone** |  |  |  |  |  |  |  |  |  |  |  |  |  |  |  |
| Linezolid | 1 | 1 | 1 |  | 1 | 1 | 1 |  | 2 | 1 | 1 |  | 1 | 1 | 1 |
| **Aminoglycoside** |  |  |  |  |  |  |  |  |  |  |  |  |  |  |  |
| Gentamicin | >8 | 4 | 8 |  | <0.25 | <0.25 | <0.25 |  | <0.25 | <0.25 | <0.25 |  | <0.25 | <0.25 | <0.25 |
| Arbekacin | 2 | 0.5 | 0.5 |  | <0.25 | <0.25 | <0.25 |  | <0.25 | <0.25 | <0.25 |  | <0.25 | <0.25 | 0.25 |
| **Tetracycline** |  |  |  |  |  |  |  |  |  |  |  |  |  |  |  |
| Minocycline | <2 | <2 | <2 |  | <2 | <2 | <2 |  | <2 | <2 | <2 |  | <2 | <2 | <2 |
| **Macrolide** |  |  |  |  |  |  |  |  |  |  |  |  |  |  |  |
| Erythromycin | 0.25 | 0.25 | 0.25 |  | >4 | >4 | >4 |  | 0.25 | 0.25 | 0.25 |  | 0.25 | 0.12 | 0.25 |
| **Lincomycin** |  |  |  |  |  |  |  |  |  |  |  |  |  |  |  |
| Clindamycin | <0.06 | <0.06 | <0.06 |  | 0.5 | 0.5 | 0.5 |  | <0.06 | <0.06 | <0.06 |  | <0.06 | <0.06 | <0.06 |
| **Fosfomycin** |  |  |  |  |  |  |  |  |  |  |  |  |  |  |  |
| Fosfomycin | <32 | <32 | <32 |  | <32 | <32 | <32 |  | <32 | <32 | <32 |  | <32 | <32 | <32 |
| **Fluoroquinolone** |  |  |  |  |  |  |  |  |  |  |  |  |  |  |  |
| Levofloxacin | 0.5 | 0.5 | 0.5 |  | 1 | 1 | 1 |  | 0.5 | 0.5 | 0.5 |  | 0.5 | 0.25 | 0.5 |
| **Sulfonamide** |  |  |  |  |  |  |  |  |  |  |  |  |  |  |  |
| Sulfamethoxazole-Trimethoprim | <10 | <10 | <10 |  | <10 | <10 | <10 |  | <10 | <10 | <10 |  | <10 | <10 | <10 |

^*^Minimum inhibitory concentration. ^†^Norgestimate. ^‡^17-deacetyl norgestimate. In these experiments, cells were cultured under biofilm-forming conditions, with 50 μM of the test compound.
