# Supplementary material for: Norgestimate inhibits staphylococcal biofilm formation and resensitizes methicillin-resistant Staphylococcus aureus to β-lactam antibiotics
Source: NPJ Biofilms Microbiomes. 2017 Jul 21;3:18. doi: 10.1038/s41522-017-0026-1 (PMC5522392; doi:10.1038/s41522-017-0026-1)
Supplement: Supplementary file 10 — Table S9. Primers for real-time PCR used in this study [file 41522_2017_26_MOESM10_ESM.docx]

Table S9. Primers for real-time PCR used in this study

| Gene | Primer (5'→3') | | Source or reference |
| --- | --- | --- | --- |
| *sasG* | Forward: | caaaagatccaaaaggacctgaa | in this study |
|  | Reverse: | ggccacttggatgagttggt | in this study |
| *eno* | Forward: | aattgaagacggtatggacgaaa | in this study |
|  | Reverse: | tcaccgatacgttctgtaagttgttt | in this study |
| *femA* | Forward: | gctggtttcttctttatcaatccatt | in this study |
|  | Reverse: | cactgcataacttccggcaaa | in this study |
| *femC* | Forward: | aacgccaaggcaaatacgtt | in this study |
|  | Reverse: | tttgaccttctgttctttcaggttt | in this study |
| *pbp1* | Forward: | cgaaagatttatttgcggttgtc | in this study |
|  | Reverse: | cgaaagatttatttgcggttgtc | in this study |
| *pbp2* | Forward: | atctggtggacgtgatttcaaa | in this study |
|  | Reverse: | ggtccatacgctaagaaaggtttta | in this study |
| *pbp3* | Forward: | aagaaatgaaatacacaacggacaaa | in this study |
|  | Reverse: | gcgagcgccaggatttaac | in this study |
| *pbp4* | Forward: | catgggtgcaggagactataaaaa | in this study |
|  | Reverse: | atgcattccccatcatattacgt | in this study |
| *atl* | Forward: | tgcagtcggtaaccctagattca | in this study |
|  | Reverse: | attgtgtagctgcatagtcagcatagt | in this study |
| *isaA* | Forward: | acagctgcgttgatttgttg | (48) |
|  | Reverse: | ctgcaggtgctactggttca | (48) |
| *lytM* | Forward: | tggtgaccaaattgcatattcag | in this study |
|  | Reverse: | cgatgccaccagacatacgt | in this study |
| *16S* | Forward: | caacgagcgcaacccttaag | (49) |
|  | Reverse: | tttgtcaccggcagtcaactt | (49) |
